# Functional consequences of a rare missense BARD1 c.403G>A germline mutation identified in a triple-negative breast cancer patient

**DOI:** 10.1186/s13058-021-01428-5

**Published:** 2021-05-01

**Authors:** Yuanting Zheng, Bingying Li, Dejing Pan, Jun Cao, Jian Zhang, Xiaolin Wang, Xiangnan Li, Wanwan Hou, Ding Bao, Luyao Ren, Jingcheng Yang, Shangzi Wang, Yangyang Qiu, Fei Zhou, Zhiwei Liu, Sibo Zhu, Lei Zhang, Tao Qing, Yi Wang, Ying Yu, Jiaxue Wu, Xichun Hu, Leming Shi

**Affiliations:** 1 State Key Laboratory of Genetic Engineering, School of Life Sciences and Shanghai Cancer Center, Fudan University, Shanghai, China; 2 Cambridge-Suda Genomic Resource Center and Jiangsu Key Laboratory of Neuropsychiatric Diseases Research, Soochow University, Suzhou, China; 3 Department of Medical Oncology, Fudan University Shanghai Cancer Center, Shanghai, China; 4 State Key Laboratory of Genetic Engineering, School of Life Sciences and Zhongshan Hospital, Fudan University, Shanghai, China; 5 Human Phenome Institute, Fudan University, Shanghai, China; 6 Fudan-Gospel Joint Research Center for Precision Medicine, Fudan University, Shanghai, China

**Keywords:** BARD1, Rare mutation, c.403G>A, p.Asp135Asn, Integrated genomics profiling, Triple-negative breast cancer, Irradiation, DNA damage, PARP inhibitor, Functional assay

## Abstract

We identified a rare missense germline mutation in *BARD1* (c.403G>A or p.Asp135Asn) as pathogenic using integrated genomics and transcriptomics profiling of germline and tumor samples from an early-onset triple-negative breast cancer patient who later was administrated with a PARP inhibitor for 2 months. We demonstrated in cell and mouse models that, compared to the wild-type, (1) c.403G>A mutant cell lines were more sensitive to irradiation, a DNA damage agent, and a PARP inhibitor; (2) c.403G>A mutation inhibited interaction between BARD1 and RAD51 (but not BRCA1); and (3) c.403G>A mutant mice were hypersensitive to ionizing radiation. Our study shed lights on the clinical interpretation of rare germline mutations of BARD1.

**Supplementary Information:**

The online version contains supplementary material available at 10.1186/s13058-021-01428-5.

Incorporation of next-generation sequencing into clinical practice continues to expand the list of variants of unknown significance (VUS), making it challenging to appropriately interpret the clinical significance of such rare mutations in terms of pathogenicity and treatment options for the patient [1–3]. Analysis of the whole-genome sequencing data from a triple-negative breast cancer (TNBC) patient’s germline DNA uncovered a c.403G>A (p.Asp135Asn) mutation in *BARD1* (Additional file 1). The BRCA1-associated ring domain 1 (BARD1) protein is a binding partner of BRCA1 and is essential for DNA damage repair [4], which is reported to be associated with breast cancer susceptibility [2, 3]. In contrast to BRCA1 and BRCA2, for which numerous pathogenic mutations and benign variants have been identified [5], validated, and used for clinical decision-making, the clinical significance of a specific BARD1 mutation remains unclear and is classified as VUS [6]. The newly identified locus is located near the ring domain of *BARD1* and highly conserved across different species (Fig. 1a). The germline variant was validated by orthogonal Sanger sequencing (Fig. 1b), and it was inherited from the father of the patient (Fig. 1c). This c.403G>A mutation was only reported in one case from 138,632 whole-exome and whole-genome sequences in the Genome Aggregation Database (gnomAD [7]) with no reports on its clinical relevance. We predicted the pathogenicity of the rare missense c.403G>A mutation using four computational tools with conflicting prediction results: tolerated by SIFT [8] and benign by PolyPhen 2 [9], while damaged by Mutation Assessor [10] and SNPs&GO [11].

**Fig. 1:**
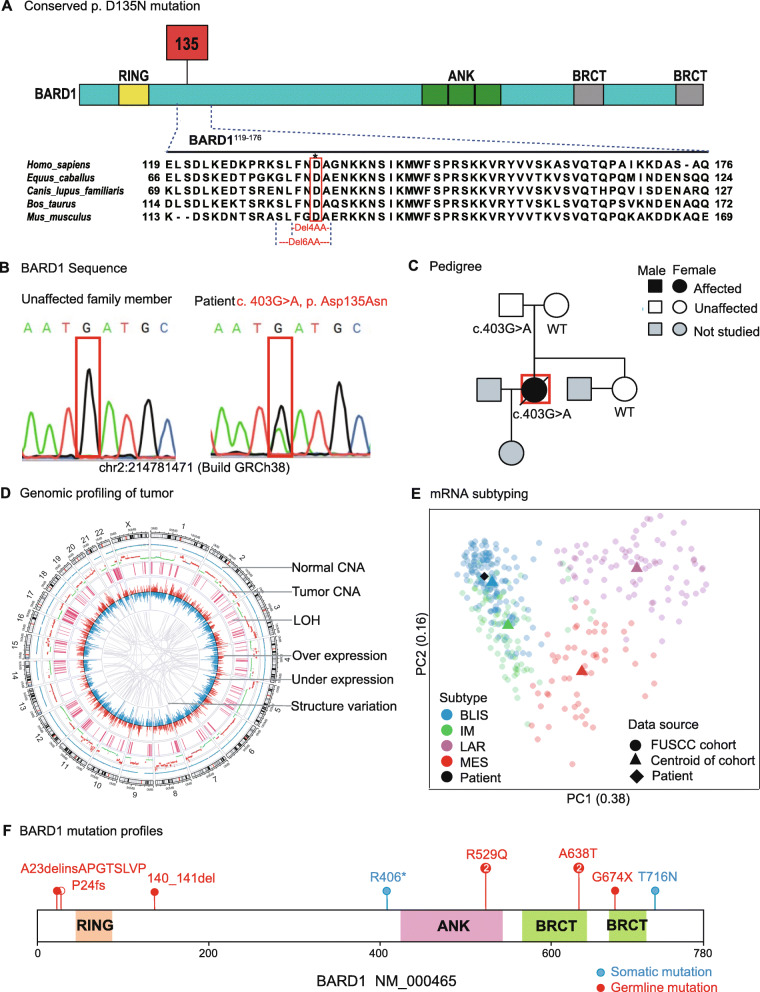
Genomic and transcriptomic profiling reveals a highly conserved, rare, and potentially pathogenic germline mutation (c.403G>A) of BARD1 in an early-onset TNBC patient that displayed a homologous recombination deficiency (HRD) somatic mutational signature. **a** A multiple sequence alignment of five mammalian BARD1 proteins. The highlighted residue 135, Asp (D), is conserved across all the species. **b** Chromatograms of the DNA sequences of the mutated locus by Sanger sequencing. **c** Genetic testing results of the patient family. **d** Genomic profiles of tumor from the patient. Large-scale copy number variations, loss of heterozygosity, and large-scale structural variations indicated an HRD somatic mutational signature. **e** The mRNA profile of the patient clustered together with the basal-like and immune-suppressed (BLIS) subgroup in a large TNBC cohort of FUSCC. **f** Somatic and germline mutation profiles of BARD1 in the FUSCC cohort. Germline variants are colored in red, and somatic mutations are colored in blue. Mutations marked with 2 indicates the mutation was detected twice in the TNBC cohort

Importantly, computational analysis of the genomics profiling from the patient’s tumor samples showed that the patient’s tumor exhibited extensive copy number changes, loss of heterozygosity, and large-scale structural variations across the entire genome (Fig. 1d), indicating a typical genomic mutational signature resulting from deficiency in homologous recombination DNA damage repair [12]. Moreover, the mRNA expression profile of the tumor clustered closely with the basal-like and immune-suppressed (BLIS) subgroup in a large cohort of 465 TNBCs from Fudan University Shanghai Cancer Center (FUSCC) (Fig. 1e) [13], a study partially inspired by the patient. In addition, the mutation profile of BARD1 in the FUSCC TNBC cohort is shown in Fig. 1f, indicating that the germline mutation rate in BARD1 is low and the mutation loci were heterogeneous. Taken together, we hypothesized that the c.403G>A mutation in BARD1 might be damaging by impairing the homologous recombination capacity of the cells.

To explore the in vitro functions of the c.403G>A (p.Asp135Asn) mutation in *BARD1*, reconstitution with wild-type *BARD1* (*BARD1*^WT^res) and mutant *BARD1* (*BARD1*^D135N^res) was conducted in two *BARD1* knockout breast cancer cells (T47D and MDA-MA-468) by the CRISPR-Cas9 system. As shown in Fig. 2a, the BARD1 protein was undetectable in the knockout cells, whereas comparable expression of the BARD1 protein was detected in rescued cells (BARD1^WT^res and BARD1^D135N^res) by Western blot using anti-BARD1 antibodies.

**Fig. 2:**
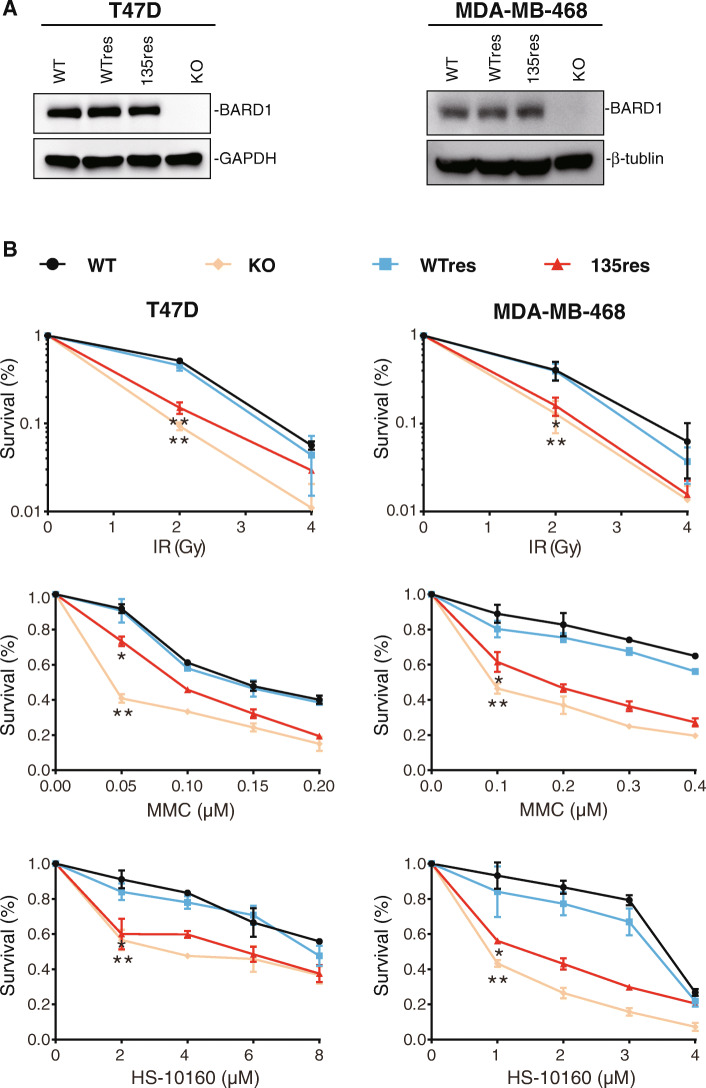
BARD1 p.Asp135Asn mutation increased cell sensitivity to DNA damage in vitro. **a** Western blot testing of the Flag-tagged BARD1 expression in BARD1 knockout (KO), wild-type BARD1^WT^ rescue (WTres), and BARD1^135^ rescue (D135Nres) breast cancer cell lines. **b** Clonogenic survival of T47D cells (left) and MDA-MB-468 (right) cells expressing BARD1^WT^res or BARD1^135^res after γ-irradiation (IR, up), and cell survival (CCK8 assay) of T47D cells (left) and MDA-MB-468 cells (right) expressing BARD1^WT^res or BARD1^135^res after treatment with mitomycin C (MMC, middle) and PARP inhibitor (HS-10160, down). Data are means ± s.d, *n*=3. *P* values were calculated using a two-tailed Student’s *t*-test. *P < 0.05, **P < 0.01

Impairment of DNA damage repair was reflected by increased sensitivity of tumor cells to DNA damage. In the γ-irradiation (IR) induced DNA damage experiment, *BARD1* knockout T47D and MDA-MB-468 cells showed significantly reduced clonogenic survival compared with control cells after irradiation (Fig. 2b, *P*< 0.01). As expected, ectopic expression of BARD1^WT^res resulted in full rescue of the reduced clonogenic survival, whereas ectopic expression of BARD1^D135N^res showed partial rescue and the clonogenic survival was still significantly reduced compared with control cells (Fig. 2b). Similar results were observed in the DNA damage experiment with DNA inter-strand crosslinking agent mitomycin C (MMC). BARD1-deficient cells expressing BARD1^D135N^res were more sensitive to MMC than cells expressing wild-type BARD1^WT^res as determined by the CCK8 cell proliferation assay (Fig. 2b). Moreover, BARD1-deficient cells expressing BARD1^D135N^res were also more sensitive to an investigational PARP inhibitor HS-10160 than cells expressing BARD1^WT^res by the CCK8 cell proliferation assays (Fig. 2b).

Mechanically, BARD1 was thought to play an important role in DNA damage repair through direct interaction with BRCA1 by forming a BARD1-BRCA1 heterodimer [14]. However, immunoprecipitation (IP) assays showed that the c.403G>A mutation in BARD1 had little effect on the interaction between BARD1 and BRCA1 (Fig. 3a). Recently, Zhao et al. reported that the BARD1 region (123-261) is indispensable for the interaction between BARD1 and RAD51, and RAD51-mediated homologous DNA pairing [15]. This raises the possibility that the p.D135N germline mutation may affect the function of BRAD1 in DNA damage repair with RAD51-mediated homologous recombination pairing. On the other hand, we found that the interaction between BARD1^D135N^res and RAD51 was significantly weaker compared to that between BARD1^WT^res and RAD51 in T47D and HEK293T cells after irradiation (Fig. 3b). Taken together, these data suggested that the c.403G>A mutation in BARD1 increased sensitivity to PARP inhibitor treatment in vitro, which may be caused by the reduction of its DNA damage-induced association with RAD51.

**Fig. 3:**
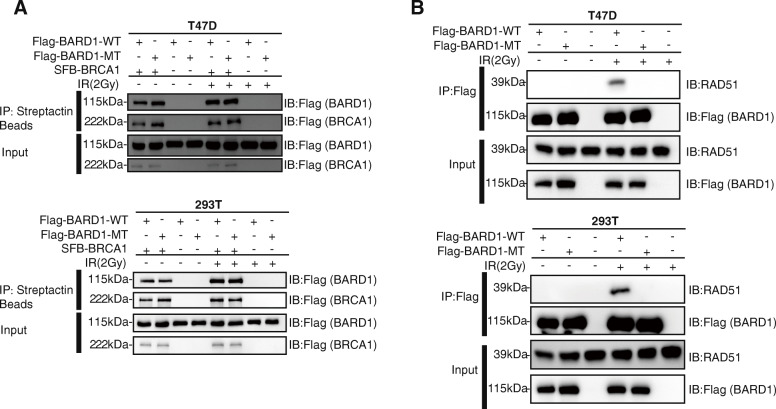
BARD1 p.Asp135Asn mutation impaired the DNA damage-induced association with RAD51 in vitro. **a** Western blot (WB) verification of the effect of BARD1MT (p.Asp135Asn) on BARD1-BRCA1 binding before and after irradiation (2 Gy). FLAG-BARD1-WT/FLAG-BARD1-MT and SFB-BRCA1 were co-transfected into T47D and HEK293T cells and applied to immunoprecipitation followed by Western blot. Whole-cell lysates were also blotted and shown as input. **b** Western blot verification of the effect of BARD1-MT on BARD1-RAD51 binding in T47D and HEK293T cells. Cells expressing BARD1-WTres and BARD1-MTres were treated with irradiation (2 Gy) and then subjected to immunoprecipitation and Western blot

To investigate the functions of the c.403G>A mutation in BARD1 in vivo, two individual mouse lines (PM#5 and PM#8) with a point mutation c.718G>A in mice (p.Asp127Asn) corresponding to the c.G403A (p.Asp135Asn) mutation in human BARD1 were obtained using the CRISPR/Cas9 system. Mice with the c.718G>A point mutation (PM#5 and PM#8) were significantly more susceptible to irradiation than the wild-type mice (Fig. 4a, b). With a sub-lethal dose (7 Gy) (i.e., 10% of the LD50 for wild-type C57BL6J male mice) of total body irradiation (TBI), the time to reach 50% lethality for wild-type control mice was nearly 4 days or longer compared to the homozygotes point mutation lines, and the *P* values of log-rank tests all showed a statistically significant difference (*P* = 0.045 for PM#5 and 0.025 for PM#8).

**Fig. 4:**
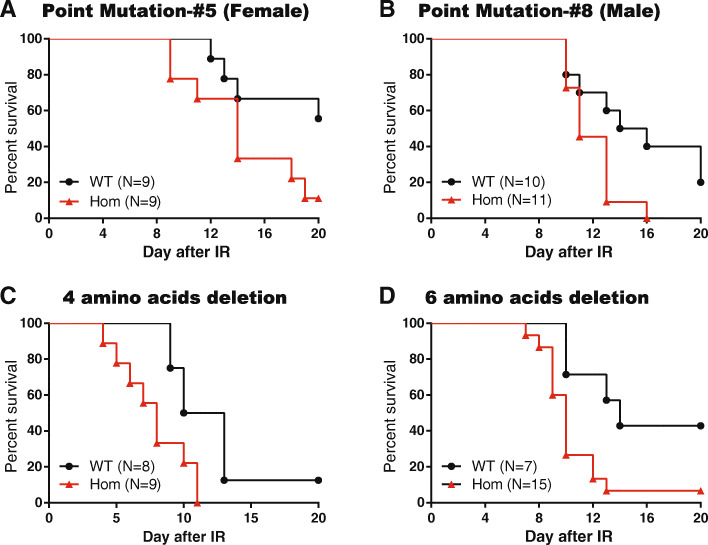
BARD1 p.Asp127Asn increased sensitivity of mice to irradiation. **a** Point mutation D127N-#5, **b** point mutation D127N-#8, **c** four amino acid deletion, and **d** six amino acid deletion were exposed to a sub-lethal dose (7 Gy) of total body irradiation (TBI). The littermate wild-type mice were used as controls. Survival at day 20 after TBI was analyzed and shown as percent mortality on the *Y*-axis. Survival curves of wild-type (WT) and homozygous mutation (Hom) mice after sub-lethal irradiation were shown. *n* ≥ 7 mice per genotype. *P* values shown are for the Wald test

Furthermore, one line with a deletion of four amino acids (FGDA) indicated as #121SRASL"FGDA"ERKKNSIKMW#138 (referred to as Del4aa) and another line with a deletion of six amino acids (SLFGDA) indicated as #121RK"SLFNDA"SRA"SLFGDA"ERKKNSIKMW#138 (referred to as Del6aa) were also obtained and undergone with the same irradiation experiments. Similarly, the deletion lines of Del4aa and Del6aa displayed a statistically significantly lower survival compared to littermate wild-type control mice (*P* = 0.0006 for Del4aa and 0.012 for Del4aa; Fig. 4c, d). This result provided strong evidence for a pivotal role of this short and conserved sequence to maintain BARD1 functions in mammals.

Taken together, both in vitro and in vivo biological validation data indicated potential clinical benefits of PARP inhibitors for patients carrying c.403G>A mutation. However, no PARP inhibitor was marketed in China and *BARD1* mutation was not used as a biomarker for enrollment of patients in clinical trials in China when the patient was diagnosed and treated. Eventually, the patient was administrated with an investigational PARP inhibitor for about 2 months as a compassionate use after oncologists exhausted all available treatment regimens. By clinical observations, the shrinkage of the primary breast tumor and the metastases to the bone and liver was obvious. No drug resistance was found during the short treatment period. Unfortunately, the patient was later on diagnosed with brain metastases, which might have already occurred before the initiation of PARP inhibitor treatment, and therefore treated with radiation therapy. However, serious anemia occurred during radiation therapy and the PARP inhibitor therapy was discontinued. It seems that incorporating radiation therapy into the treatment scheme was not advisable in such a situation due to increased sensitivity resulting from the rare germline mutation.

Our results added evidence that the inherited c.403G>A mutation in the highly conserved functional domain of BARD1 appears to suggest a favorable response of a triple-negative breast cancer patient to a PARP inhibitor, thus benefiting patients beyond carriers of BRCA1 or BRCA2 germline mutations. Incorporation of next-generation sequencing into clinical practice continues to expand the list of VUS in DNA damage repair genes, posing particular challenges in the clinical decision for carriers of the VUS about personalized drug therapy and genetic counseling. Our integrated approach by combining the identification of germline mutation with somatic and transcriptomic profiling of cancer patients, followed by functional assays at the molecular, cellular, and animal levels, will accelerate the interpretation of VUS for precision medicine.

## Supplementary Information

**Additional file 1. **Study case, sample preparation, data generation, bioinformatics analyses, cell culture assays, development of mouse models with specific mutations, and *in vivo* irradiation assays with mutant mice were performed as described.

## Data Availability

The datasets used and/or analyzed during the current study are available from the corresponding authors on reasonable request.

## Abbreviations

HR: Homologous recombination
IR: γ-irradiation
MMC: Mitomycin C
PARP: Poly ADP-ribose polymerase
VUS: Variants of unknown clinical significance
WT: Wild-type
