# Supplementary material for: Functional consequences of a rare missense BARD1 c.403G>A germline mutation identified in a triple-negative breast cancer patient
Source: Breast Cancer Res. 2021 May 1;23:53. doi: 10.1186/s13058-021-01428-5 (PMC8088670; doi:10.1186/s13058-021-01428-5)
Supplement: Supplementary file 1 — Additional file 1. Study case, sample preparation, data generation, bioinformatics analyses, cell culture assays, development of mouse models with specific mutations, and in vivo irradiation assays with mutant mice were performed as described. [file 13058_2021_1428_MOESM1_ESM.docx]

**Additional file 1**

**METHODS**

**Study case**

A 28-year-old female was diagnosed with stage IV triple-negative breast cancer (TNBC) with bone and liver metastases in Changsha, Hunan Province, China in December 2014. The patient was referred to Fudan University Shanghai Cancer Center (FUSCC) in May 2015 after having been treated with chemotherapy (5 cycles of TAC, 1 cycle of gemcitabine + carboplatin) for 4 months in Changsha, where her physicians had exhausted treatment options. The patient was immediately tested for germline *BRCA1/2* mutations hoping to be eligible for enrollment in an AstraZeneca-sponsored clinical trial of olaparib (AZD-2281), a PARP (poly ADP-ribose polymerase) inhibitor for ovarian and breast cancer. Disappointedly, the patient was determined to be ineligible for the olaparib trial because of the lack of germline *BRCA1/2* mutations.

In order to identify actionable variants for drug treatment selection, we conducted extensive omic profiling including whole-genome sequencing (WGS), CytoScan^®^ HD array for copy number variation, and RNA-seq analyses of cone-needle primary breast tumor biopsies and paired normal breast tissues from the patient. We also validated the rare germline variant found in the patient and her family members using Sanger sequencing.

**Sample Processing**

Both tumor and matched normal breast tissues were collected using cone needles. DNA was extracted for genomic profiling using DNeasy blood & tissue kit (Qiagen, Hilden, Germany) according to the provider’s protocols. Total RNA was extracted using a MiRNeasy mini kit (Qiagen, Hilden, Germany).

**Whole-Genome Sequencing and Data Analysis**

Two hundred (200) ng of DNA sample was fragmented and used for library preparation by TruSeq Nano DNA library kit (Illumina, San Diego, CA, USA) according to the manufacturer’s protocols. The libraries were sequenced on an Illumina HiSeq XTEN platform (Illumina Inc., San Diego, CA, USA).

The average coverage of samples was 30X. The whole-genome sequenced reads were aligned to human genome (hg19) with BWA-mem, and the resulting BAM files were preprocessed using Sentieon tools version 201711 (https://www.sentieon.com/). Somatic variant calling was conducted using TNseq (sentieon driver -r --algo TNhaplotyper --dbsnp --cosmic, https://www.sentieon.com/) and TNscope (sentieon driver -r --algo TNscope --dbsnp --cosmic; https://www.sentieon.com/) for single nucleotide variants (SNVs) and indels based on paired tumor and normal samples, as previously reported (1). Germline variant calling was conducted using TNseq (sentieon driver -r --algo TNhaplotyper–d; <https://www.sentieon.com/>) for single nucleotide variants (SNVs) and indels based on normal sample. Structural variation was analyzed using Delly v0.7.7 using default parameters (2). Mutations were annotated using ANNOVAR (3). Sequencing quality statistics were calculated using FastQC (<https://www.bioinformatics.babraham.ac.uk/projects/fastqc/>), Qualimap (4), and MuiltiQC (5).

**RNA Sequencing and Data Analysis**

Total RNA (1 µg) spiked with 2 µL 1:100 diluted ERCC RNA spike-in control mix 1 or mix 2 (Life Technologies, Carlsbad, CA, USA) was depleted of rRNA with the Ribo-Zero protocol (Illumina, San Diego, CA, USA) and then sequenced on an Illumina HiSeq platform. Paired-end reads with lengths of 150 nucleotides were generated.

After quality control process and trimming using Trimmomatic (6), reads were aligned to a human reference genome (hg19) and quantified using the tophat-cufflinks pipeline (7). FPKM (Fragments Per Kilobase of exon model per Million mapped fragments) values of tumor and normal tissues were obtained for further analysis.

**Generation and Analysis of Copy Number Alteration Array Data**

Genome-wide copy number arrays (Affymetrix, Santa Clara, CA, USA) analysis of tumor and matched normal breast tissues were conducted using a CytoScan HD array assay kit (Affymetrix, Santa Clara, CA, USA) according to the manufacturer’s protocols. The arrays were scanned with a GeneChip Scanner 3000 7G (Affymetrix, Santa Clara, CA, USA). CEL files were used for analysis with OncoScan Console v1.3 software (Affymetrix, Santa Clara, CA, USA). Probe-level copy number value and gene-level loss of heterozygosity score (LOH) was obtained from the OncoScan Console.

A circos plot was drawn using the circlize package (8) to present an overview of the omic profiles across the genome, including copy number alterations, LOH, differential gene expression, and structural variations.

**Dataset of a Chinese TNBC cohort**

Somatic and germline mutation profiles across patients were obtained from Jiang *et al*. (1), a cohort of 465 Chinese TNBC patients. Among these 465 patients, 279 had whole-exome sequencing (WES) data on primary tumor tissue and paired normal samples, 52 had WES data on blood samples.

Germline variations were obtained from 331 normal samples from the TNBC patients. Mutations with population allele frequency lower than 1% in East Asia in 1000G, ExAC, gnomAD and ESP6500 databases were remained for further analysis.

In addition, gene-expression data were generated using primary tumor tissues from 360 patients in the cohort. Samples were clustered into four subtypes based on expression profiles as previously described (1). Two hundred and five (205) differentially expressed genes across the four subtypes were selected using the cutoff of a t-test *p* value <0.05 and fold change (FC) ≥2 or ≤0.5. Centroid values across the 205 genes in each subgroup were used as a biomarker for decision making. In order to classify a sample into one of the four subgroups, we calculated the Spearman correlation coefficient between the expression profile of the 205 genes of the sample and that of the centroid of four subgroups, and a sample is classified into the subgroup with the highest correlation coefficient. Principal component analysis (PCA) was performed for classification and visualization.

**Sanger sequencing**

The BARD1 p.Asp135Asn (g.215646195C>T, c.403G>A) was confirmed by polymerase chain reaction (PCR). The following primers were used: primer forward (5’- GGCACAAAGTCGGATTCAGT-3’) and primer reverse (5’-AACATCTGCAGGAGGACTTG-3’) for the allele. Reactions were performed in a 50 µL reaction mixture containing 40 ng of DNA, 200 nM primer, 0.6 mM MgCl_2_, 0.1 mM each dNTP, and 1 unit of KOD FX Taq DNA polymerase (Toyobo, Osaka, Japan). The cycling conditions were 4 min at 94°C (one cycle); 10 sec at 98°C, 30 sec at 59°C, and 1 min at 68°C (31 cycles); and 5 min at 72°C (one cycle). PCR fragments with the presence of a 404 bp band were sent for Sanger sequencing.

**Plasmid construction**

SFB-BRCA1 (BRCA1-pIRES-EGFP, S-Flag-SBP (Streptavidin Binding Peptide)-tagged (SFB tagged)) was a gift from the laboratory of Prof. Jiaxue Wu. Human BARD1 (NCBI Accession number NP_000456.2) was cloned into modified pcDNA3.1 and pLVX-Puro to generate constructed encoding triple Flag tagged BARD1. Quick change mutagenesis (Stratagene, La Jolla, CA, USA) was conducted to generate the point mutants of BARD1 using oligos 1 (5′-GTTTGTTTAATAACGCAGGAAACAAGAAGAATTCAA-3′) and 2 (5′-TTGTTTCCTGCGTTATTAAACAAACTTTTCCTAGGT-3′). sgRNAs (5ʹ-GCGGGACGATGCCGGATAAT-3ʹ) that targeted BARD1 exon 1 were designed via http://crispr.mit.edu website and subcloned into a pX335-U6-Chimeric-BB-CBh-hSpCas9n vector.

**Cell culture and transfection**

T47D and MDA-MB-468 human breast cancer cells obtained from ATCC were maintained in RPMI-1640 medium (Gibco, Thermo Fisher Scientific Inc., Waltham, MA, USA) supplemented with 10% fetal bovine serum (FBS; Gibco, Thermo Fisher Scientific Inc., Waltham, MA, USA), 100 U/mL penicillin and streptomycin. Cell line authentication were performed by Shanghai Genechem Co., Ltd using Short Tandem Repeat (STR) analysis as described in 2012 in ANSI Standard (ASN-0002).Cells were cultured in an incubator at 37°C supplemented with 5% CO_2_. For transfection, HEK293T or breast cancer cells at 70% confluence were transfected with plasmids using Lipofectamine (Invitrogen, Carlsbad, CA, USA) according to the manufacturer’s recommended protocol.

**Generation of BARD1-deficient and BARD1-reconstituted cells**

BARD1-deficient cells were generated using the CRISPR-Cas9 system. A constructed vector that targeted exon 1 of BARD1 was transiently transfected into T47D and MDA-MB-468 cells. After 48 h, the transfected cells were selected with puromycin for 3 days, and single colonies were screened through Western blot by using the anti-

BARD1 antibody. The sgRNA-targeting genomic regions of positive clones were amplified and sequenced.

BARD1-reconstituted cells were established through lentivirus infection. In brief, wild-type or inactive mutant BARD1 lentivirus was packaged by the co-transfection of PLVX-BARD1-WT or PLVX-BARD1-135 with pCMV-dR8.2 dvpr and pCMV-VSVG into HEK293T cells. After 48 h, the cell culture medium was collected in accordance with the manufacturer’s protocols to obtain the packaged lentivirus. BARD1-deficient T47D and MDA-MB-468 cells were infected with BARD1^WT^ or BARD1^135^ lentivirus and verified through Western blot by using the indicated antibodies.

**Radiation treatment and colony formation assay**

T47D and MDA-MB-468 cells (1000 cells/well) were plated into a 6-well plate. Twelve hours later, cells were treated with 2 Gy of Co-60. Media were changed two hours later and cultured for 14-28 days. Colonies on the plates were then fixed with 4% formaldehyde and stained with 0.1% crystal violet (Sigma-Aldrich, St. Louis, MO, USA). Surviving colonies with more than 50 cells were counted and the surviving percentage was calculated and normalized with parental cells by Image J (Bethesda, MD, USA).

**Cell viability assay**

Cells (1000 cells/well) were seeded in a 96-well plate and cultured for 24 hours. Subsequently, cells were treated with mitomycin C (MMC, Meilunbio, China) and PARP inhibitor HS-10160 (Hengrui Medicine, Shanghai, China) under different concentrations of 0, 0.05, 0.1, 0.15, and 0.2 nm/mL. Viable cells were detected using CCK-8 (Cell Counting Kit-8, Dojindo Kagaku Co, Kumamoto, Japan) according to the manufacturer’s protocols. After 72 h, supernatants were replaced by CCK-8 solution and cells were incubated for another 2 h at 37°C. The optical density (OD) value for each well was read at 450 nm and 630 nm using an automated microplate reader and calculated with the GraphPad Prism 5 software (GraphPad Software, La Jolla, CA, USA).

**Co-IP**

SBP (Streptavidin Binding Peptide) or FLAG–tagged protein was immunoprecipitated by using Streptactin Beads 4FF (Smart, Shanghai, China) or Anti-FLAG® M2 Magnetic Beads (Sigma-Aldrich, St. Louis, MO, USA) following the manufacturer’s instructions. In the Co-IP experiment for BRCA1 and BARD1, HEK293T and T47D cells were collected 24 hours after transfection with indicated plasmids (FLAG-BARD1WT, FLAG-BARD1MT (p.Asp135Asn) and SFB-BRCA1). In the Co-IP experiment for RAD51 and BARD1, T47D and HEK293T cells were treated with 2 Gy of IR and incubated at 37°C for 8 hours before collection.

Collected cells were then lysed on ice for 10-15 minutes in NETN buffer (20 mM Tris-HCl pH 8.0, 100 mM NaCl, 1 mM EDTA, and 0.5% Nonidet P-40). Then the whole cell lysates were centrifuged at 12,000 x g, for 10 min at 4°C, followed by collection of supernatants. 30 μL of agarose beads were add to the supernatants and incubated at 4°C for 3 h. After incubation, pellets were washed by NETN buffer for 5 times and boiled in SDS loading buffer for 20 minutes. Then immunoprecipitated proteins were analyzed by Western blot with indicated antibodies.

**Generation of BARD1-mutant mice**

Specific pathogen free (SPF) mice were maintained in accordance with the IACUC of Cambridge-Suda Genomic Resource Center of Soochow University (Suzhou, China). All mice used during this study were kept with a 12:12-h light:dark cycle. The experiments were carried out under certified animal protocol CAM-SU-AP#DP2018-001.

The human BARD1 p.Asp135Asn mutation was mimicked in mouse. The human BARD1 p.Asp135 amino acid (aa) is located as shown in Bold/Italic letter in “RASLFG***D***AERKKNSIKMW”, and its counterpart in mouse (Asp127) is shown in **Bold**/*Italic* letter in “RKSLFN***D***AGNKKNSIKMW”. Two targeted sgRNA sequences were chosen via “crispr.mit.edu” as “ACATCTAGGGCAAGTTTATT” and “GTTTATTTGGTGATGCAGAA”. ssDNA: *atccttatagcatgtttcttttccttttttctatcag*ATTCAAAAGACAAC ACATCTAGGGCtAGcTTgTTTGGTaAcGCtGAgAgaAAGAAGAATTCAATA AAAATGTGGTTTAGTCCTCGAAGTAAGAAGGTTAGATATGTTGTGACT (Intron: Italic, low case letters were used for synonymous mutation). “aAc” is designed for mutation of “GAT” to fulfill the D to N amino acid mutation.

C57BL/6J and ICR mouse strains were used as embryo donors and foster mothers, respectively. Super ovulated female C57BL/6J mice were mated to C57BL/6J males, and zygotes were collected from oviducts at E0.5. Cas9 mRNA (25 ng/µL), single sgRNA (30 ng/µL), and ssDNA (2 µM) in 20 µL were microjected into the cytoplasm of zygotes with well recognized pronuclei. Injected zygotes were cultured to blastocysts for genomic analysis or transferred at 2-cell stage into the oviduct of pseudopregnant ICR female mice to generate offsprings.

***In vitro* transcription**

The Cas9 plasmid was transcribed *in vitro* using T7 ULTRA (Ambion, AM1345), and its mRNA was purified using RNeasy Mini Kit (QIAGEN, 74104). sgRNA oligos were amplified with a template of pGL3-U6-sgRNA-PGK-Puro with T7 promoter (Addgene, 51133), and transcribed using MEGAshorttranscript T7 KIT (Ambion, AM1345). The sgRNAs were purified using MEGAclear Kit (Ambion, AM1908).

**Irradiation of the mice**

In the morning of study Day 0, mice with age ranging from nearly 2 to 6 months were exposed to a uniform of the total body irradiation (TBI) dose of 700 cGy X-ray radiation at 2-hour intervals with an exposure rate of 150 cGy/minute (Model X-RAD320iX, Precision X-Ray, North Branford, Connecticut, USA).

**Health status monitoring**

Irradiated mice were observed for morbidity or mortality daily and scored a scale of zero to three for signs meeting the criteria for early euthanasia based on three parameters: hunched posture, eyes appearance, and activity. Mice with scores of eight or nine underwent euthanasia by cervical dislocation.

**Statistics**

Experimental data were collected from experiments which were repeated at least three times and calculated with the GraphPad Prism 5 software (GraphPad Software, La Jolla, CA, USA). Biostatistical comparisons between experimental groups were analyzed by a paired 2-tailed Student’s t test, and the *P<0.05,**P<0.01, and ***P<0.001 were considered as statistical signiﬁcance levels.

**REFERENCES**

1. Jiang YZ, Ma D, Suo C, Shi J, Xue M, Hu X, et al. Genomic and Transcriptomic Landscape of Triple-Negative Breast Cancers: Subtypes and Treatment Strategies. Cancer Cell. 2019;35(3):428-40 e5.
2. Rausch T, Zichner T, Schlattl A, Stutz AM, Benes V, Korbel JO. DELLY: structural variant discovery by integrated paired-end and split-read analysis. Bioinformatics. 2012;28(18):i333-i9.
3. Yang H, Wang K. Genomic variant annotation and prioritization with ANNOVAR and wANNOVAR. Nat Protoc. 2015;10(10):1556-66.
4. Okonechnikov K, Conesa A, Garcia-Alcalde F. Qualimap 2: advanced multi-sample quality control for high-throughput sequencing data. Bioinformatics. 2016;32(2):292-4.
5. Ewels P, Magnusson M, Lundin S, Kaller M. MultiQC: summarize analysis results for multiple tools and samples in a single report. Bioinformatics. 2016;32(19):3047-8.
6. Bolger AM, Lohse M, Usadel B. Trimmomatic: a flexible trimmer for Illumina sequence data. Bioinformatics. 2014;30(15):2114-20.
7. Trapnell C, Roberts A, Goff L, Pertea G, Kim D, Kelley DR, et al. Differential gene and transcript expression analysis of RNA-seq experiments with TopHat and Cufflinks. Nat Protoc. 2012;7(3):562-78.
8. Gu Z, Gu L, Eils R, Schlesner M, Brors B. circlize Implements and enhances circular visualization in R. Bioinformatics. 2014;30(19):2811-2
